# Exosomes as crucial emerging tools for intercellular communication with therapeutic potential in ovarian cancer

**DOI:** 10.2144/fsoa-2022-0032

**Published:** 2023-03-06

**Authors:** Shika Hanif Malgundkar, Yahya Tamimi

**Affiliations:** 1 Department of Biochemistry, College of Medicine & Health Sciences, Sultan Qaboos University, PO Box 35, PC 123, Muscat, Sultanate of Oman

**Keywords:** biomarkers, cell-free messengers, exosomes, molecular cargo, ovarian cancer

## Abstract

More than two-thirds of epithelial ovarian cancer (EOC) patients are diagnosed at advanced stages due to the lack of sensitive biomarkers. Currently, exosomes are intensively investigated as non-invasive cancer diagnostic markers. Exosomes are nanovesicles released in the extracellular milieu with the potential to modulate recipient cells' behavior. EOC cells release many altered exosomal cargoes that exhibit clinical relevance to tumor progression. Exosomes represent powerful therapeutic tools (drug carriers or vaccines), posing a promising option in clinical practice for curing EOC in the near future. In this review, we highlight the importance of exosomes in cell–cell communication, epithelial–mesenchymal transition (EMT), and their potential to serve as diagnostic and prognostic factors, particularly in EOC.

## Plain language summary

Exosomes are nanovesicles released in the extracellular milieu by diverse cell types and serve as carriers of proteins, and nucleic acids, with the potential to modulate the recipient cells. Understanding the functions of exosomes as messengers between cancer and healthy cells unfolds new avenues in intercellular signaling mechanisms. In this review, we have highlighted the importance of exosomes in cell–cell communication and their potential to serve as diagnostic and prognostic factors in EOC. Furthermore, we believe that exosomes could represent a powerful therapeutic tool due to their ability to function as drug carriers or vaccines posing a promising option in curing EOC.

## Graphical abstract

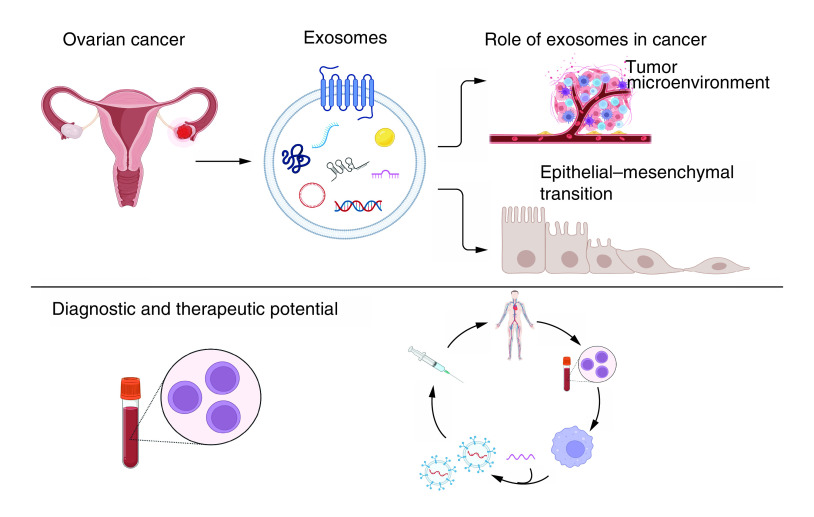

Ovarian cancer is a lethal gynecological malignancy and is commonly regarded as a silent killer due to the lack of symptoms in the early stages of the disease [1,2]. Epithelial ovarian cancer (EOC) arises from the malignant transformation of the cells in the epithelial layer of the ovary, accounting for 90% of the OC cases and is considered the most malignant subtype [3–5], while stromal and germ cell tumors constitute 6% and 4% of ovarian cancer, respectively [6]. EOC represents the 8th most common cancer among women worldwide [7], accounting for 3.4% of cancer cases [7]. In 2020, almost 4.4 million cancer-related deaths were recorded worldwide, and 4.7% of the mortality cases were attributed to EOC [7].

Stage I EOC patients account for only 10% of the total EOC cases and are characterized by a more favorable prognosis [8,9]. However, most EOC patients are diagnosed at the late stages (stages III and IV) due to the absence of reliable early-stage biomarkers [10–12]. Currently, the most commonly used biomarker for detecting EOC is serum cancer antigen (CA125). However, serum CA125 is over expressed in benign conditions such as ovarian cysts and endometriosis and is usually detected in the late stages [13–16] Thereby CA125 lacks specificity and sensitivity. Similarly, another approved EOC biomarker, Human epididymis secretory protein E4 (HE4), is not detectable in the asymptomatic and early stages of EOC [17–19]. Hence, the lack of efficient diagnostic/prognostic markers along with resistance to drug treatment and recurrence are the leading causes of poor overall survival [20]. Patients diagnosed with low-grade EOC have a five-year survival rate of >95%, while those diagnosed with high-grade EOC have a five-year survival rate ranging between 20 to 40% [21,22]. Understanding the mechanism underlying ovarian cancer pathogenesis and identifying a panel of tumor biomarkers is essential for screening, diagnosing, and treating EOC [23] and better survival outcomes [24–26].

Exosomes have recently been implicated in a plethora of physiological and pathological processes, including cancer [23,27]. Furthermore, numerous studies have reported the importance of exosomes in EOC due to their role in early diagnosis, prognosis, chemoresistance, targeted therapy, and their ability to serve as signaling molecules between the tumor cells and surrounding cells [28]. Further elucidation of the link between EOC and exosomes is crucial for uncovering the mechanisms of EOC progression and designing novel therapeutic strategies [27]. In this review, we aim to highlight the involvement of exosomes in intercellular communication, describe the molecular contents of exosomes focusing on the noncoding RNAs (ncRNAs), clarify the role of exosomal contents in affecting critical molecular processes such as epithelial to mesenchymal transition (EMT), in EOC (the most common type of OC). In addition, we also aim to shed light on the potential application of exosomes as biomarkers in EOC and discuss future therapeutic strategies.

## Overview on exosomes

Exosomes are a subset of extracellular vesicles (EV) composed of a lipid bilayer ranging from 30–100 nm in diameter [29]. It is released by several cell types, such as red blood cells, lymphocytes, dendritic cells, platelets, and tumor cells [30]. It has been detected in various biological fluids, including blood plasma, saliva, nasal secretion, cerebrospinal fluid, urine [30], and ascites [31], suggesting its role in modulating physiological responses [32]. Exosomes were earlier identified as a tool to eliminate cellular waste [33,34]. However, with advancements in current research, exosomes have been revealed to serve as carriers of a complex mixture of relevant biological molecules, including lipids, proteins, microRNAs (miRNAs), and long noncoding RNAs (lncRNAs), some of which have the potential to serve as a biomarker [35]. Furthermore, exosomes transport their cargo to the encountered recipient cells, with the potential to reprogram these target cells [36].

## Exosomes: biogenesis & composition

### Biogenesis

The biogenesis of exosomes involves early endosome formation through the invagination of the plasma membrane [37,38], a fusion of the early endosomes with the Golgi networks [39], inward budding of the endosomal membrane to form intraluminal vesicles (ILVs) known as multivesicular bodies (MVB), and the fusion of the MVB with the plasma membrane to release the exosomes [37,38]. Initially, the plasma membrane internalizes to form an early endosome [35] that matures into late endosomes (MVB) [40]. The members of the endosomal sorting complex required for transport mechanisms (ESCRT family; a cytoplasmic multi-subunit complex), such as tumor susceptibility gene 101 (TSG101) and apoptosis-linked gene 2 interacting protein X (Alix), are involved in this process and are crucial components of exosomes [41]. These MVBs enclose the intraluminal vesicles (ILV), which are formed by the inward budding of the membrane in their lumen, thereby sequestering proteins, lipids, and nucleic acid [40].

The biogenesis of ILVs and MVBs occurs through two approaches: a mechanism involving ESCRT and ESCRTs-independent mechanism. The ESCRT protein machinery involves ESCRT-0, ESCRT-I, ESCRT-II, and ESCRT-III, in addition to proteins such as Alix and TSG101. The ubiquitinated contents recruit the ESCRT-0 complex to the membrane of the endosomes. Subsequently ESCRT-I and ESCRT-II promote membrane deformities. The MVBs are isolated from the cytoplasmic membrane by the ECRT-III [42]. MVBs are transported to the cell membrane through the cytoskeleton and microtubule network, followed by the fusion with the cell membrane to release the exosomes into the extracellular space [35,40]. The Rab GTPases are responsible for the secretion of the exosomes as well as the recycling of the endosomes [43]. Depending on the parental cell and the cargoes, the regulation of exosome secretion by the Rab GTPases is heterogeneous. Soluble N-ethylmaleimide-sensitive component attachment protein receptor (SNARE) proteins are also involved in the exosome secretion [44]. Furthermore, precise targeting of the exosomes to the recipient cells is ensured by the surface components of the exosomes [45]. The released exosomes then interact with the recipient cells through several different mechanisms such as fusion with the plasma membrane to discharge their cargoes into the cytosol, stimulation of intracellular signaling *via* direct signaling through surface molecules, receptor-mediated endocytosis, or phagocytosis [46,47].

## Composition

**Figure 1.**
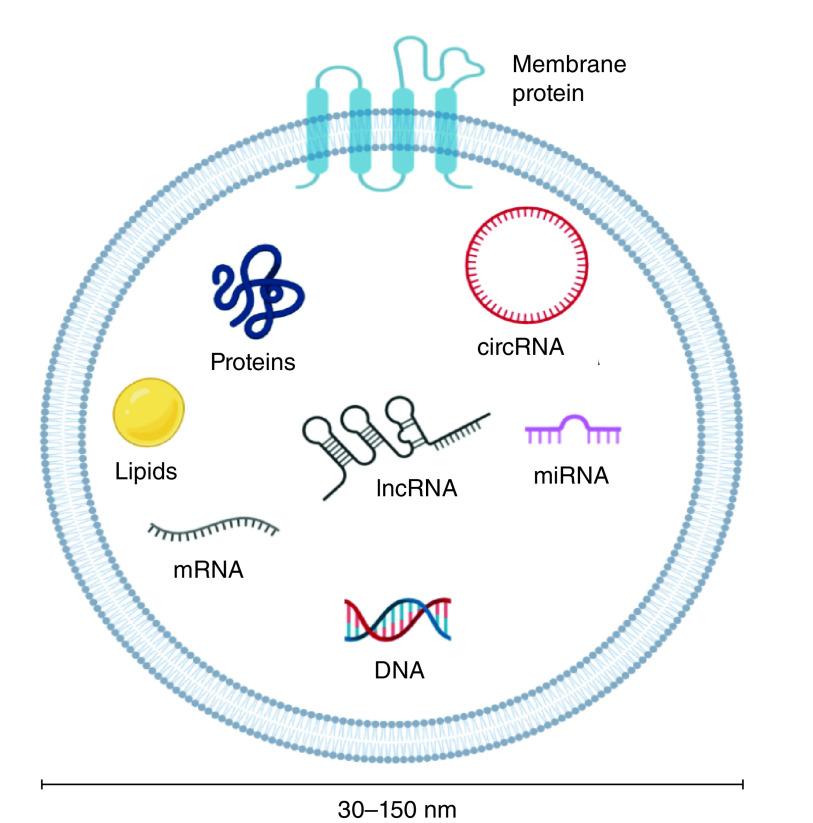
Representation of the structure of exosomes enclosing nucleic acids, and proteins as potential biomarkers in cancer. Created with BioRender.com.

The molecular contents enriched within the exosomes strongly reflect the parental cell [41], which vary according to the cellular origin, developmental stages, environmental conditions, and epigenetic changes [32]. In addition to messenger RNA (mRNA) and DNA, several other RNA species such as miRNAs, ribosomal RNA (rRNAs), transfer RNAs (tRNAs), or lncRNAs are enriched within the exosomes. Lipids such as cholesterol, phospholipids, sphingolipids, and ceramide form the bilayer membrane structure of the exosomes [41,48]. Interestingly, exosomes carry distinct proteins such as ESCRT-I associated protein (TSG101), MVB associated protein (Alix), tetraspanins (CD9, CD63, CD81, and CD82) exclusively associated with the biogenesis of the exosomes and are used as markers for their authentication (see Figure 1) [46]. Furthermore, Ras-related proteins (Rabs), membrane-binding proteins (annexins), and major histocompatibility molecules are found in the exosomes [49].

## Function of exosomes in the tumor microenvironment

Intercellular communication is crucial for maintaining physiological homeostasis and pathological processes [50]. The development and progression of cancer require intercellular communication within the primary tumor microenvironment (TME) and at distant sites [51]. Hence, TME has a crucial function in influencing the hallmarks of cancer [52,53]. TME is composed of nonmalignant cells, blood vessels, stromal cells (fibroblasts, mesenchymal stem cells (MSCs), endothelial cells, adipocytes, and lymphatic network), immune cells (natural killer cells, B and T lymphocytes, and tumor-associated macrophages), exosomes, tumor cells and extracellular matrix [54–56]. Exosomes serve as signaling molecules by transferring bioactive molecules between the cancer cells and the surrounding cells [57], thereby modifying the TME to affect the normal cells. Hence, exosomes are crucial for linking healthy cells to cancer cells.

Furthermore, exosomes derived from tumor play a key role in the crosstalk between the tumor microenvironment and cancer cells by facilitating epithelial-to-mesenchymal transition (EMT), migration, invasion, angiogenesis, immune regulation, and stromal reprogramming to a cancer-associated phenotype within the TME [58,59]. Therefore, exosomes have a profound impact on the TME [60]. Furthermore, EVs derived from tumor cells were found to influence the stromal cells, such as fibroblasts and endothelial cells, within the tumor microenvironment [50], thereby engaging in crosstalk for sustaining the signaling networks for cancer progression [61]. In order to promote metastasis, stromal cells such as a tumor associated macrophages (TAMs), cancer-associated fibroblasts (CAFs), and MSCs secrete cytokines and exosomes that induce cell proliferation, EMT, and the extracellular matrix (ECM) remodeling [62]. In addition, CAFs play a key role in initiating an inflammatory process and suppressing the activity of cytotoxic T lymphocytes [63].

## EOC-derived exosomes play a key role in modulating TME

The remodeling of ECM is a crucial step in facilitating the invasiveness of the tumor [50]. Recent studies have shown the role of cancer cell-derived exosomes in organizing the ECM. The exosomes released by the cancer cells are enriched with proteases, which can modify the ECM by degrading collagen, laminin, and fibronectin resulting in tumor and host cell adhesion, metastasis, and invasiveness [64]. In a previous finding, Rab27B regulated the release of HSP90 enriched exosomes from metastatic breast cancer cells to activate matrix metalloproteinase 2 (MMP2), thereby degrading the components of ECM and facilitating the secretion of growth factors leading to invasion [65].

The interactions between exosomes from EOC cells and TME has a pivotal role in cancer (see Table 1) [66].

**Table 1.** Influence of OC derived exosomes on endothelial, stromal, and immune cells within the TME.

Exosomal content	Target cell	Target gene	Role	Ref.
*lncRNA ATB*	HUVEC	*miR-204-3p/TGF*_*β*_R2	Promoted angiogenesis	[67]
*miR-205*	HUVEC	*PTEN*	Promoted angiogenesis *via* PTEN/AKT pathway	[68]
*lncRNAs* *(ENST00000444164* *ENST0000043768)*	HUVEC	-	Promoted migration of endothelial cells	[69]
*miR-124*	Normal fibroblasts	*Sphingosine kinase 1 (SPHK1)*	Inhibited the transition of normal fibroblasts to CAF	[70]
*miR-222-3p*	Macrophages	*SOCS3*	Activates macrophages to a tumor associated macrophage like phenotype	[71]
*miR-21-3p* *miR-125b-5p* *miR-181d-5p*	Macrophages	*SOCS4/5/STAT3*	Induction of non-polarized macrophages to tumor associated macrophage phenotype	[72]

In EOC, lymphatic dissemination is preceded by peritoneal dissemination, a specific form of metastasis. Peritoneal dissemination is a major obstacle in treating OC. A better understanding of the functions of exosomes in facilitating peritoneal dissemination could result in the better management of EOC. Exosomes facilitate peritoneal dissemination through cross-talks between the cancer cells and the tumor microenvironment [73–75]. Malignant ascites are common in OC patients and are characterized by high levels of tumor-enhancing factors such as IL-6, IL-8, IL-10, TGFβ, and vascular endothelial growth factor (VEGF). Furthermore, malignant ascites promote OC cell proliferation, anti-apoptosis, invasion, and chemoresistance *via* these factors [76–78].

## Crucial regulatory role of exosomes in the EMT process

Metastasis is a primary reason for cancer-related deaths [79] and is induced by the cancer cells undergoing EMT [80]. EMT is a process by which epithelial cells lose cell polarity and adhesive properties and attain migratory and invasive characteristics, promoting malignancy [81]. The exosomes originating from tumor cells carry EMT-inducing molecules such as the developmental transcription factors (SNAIL/SLUG, TWIST1/TWIST2, and ZEB1/ZEB2 family) [82], TGF-β, miRNAs, β-catenin, or hypoxia-inducible factors. These factors can confer mesenchymal characteristics to epithelial cells, promoting transformation and metastasis [58].

Recently, EMT was described to progress through a spectrum of stable hybrid states, namely intermediate EMT, partial EMT (pEMT), hybrid epithelial/mesenchymal, and EMT-like states [83–85]. pEMT is characterized by the partial loss of epithelial markers and gain of mesenchymal markers and has been revealed to modulate the hallmarks of cancer [86]. In addition to paracrine signaling *via* the stromal cells in the TME, genetic, epigenetic, and post-translational modifications in the primary tumor cells are responsible for inducing the pEMT cell state [83,87,88]. In OC, pEMT cell states confer more malignancy than mesenchymal phenotype and may promote ascites formation and peritoneal metastasis [89]. Exosomes are known to regulate the phenotypic pEMT by altering the transcription factors to promote invasiveness and chemoresistance [90].

The reversal of mesenchymal phenotype to epithelial state is termed mesenchymal-epithelial transition (MET), which commonly occurs during cellular development and cancer metastasis [91]. In OC, *miRNA-1294* promoted MET by suppressing *insulin-like growth factor 1 receptor* (*IGF1R*), an anti-apoptotic gene, thereby inhibiting AKT, mTOR, and ErbB [92]. Similarly, in liver cancer, *Phosphatase of regenerating liver-3* (*PRL-3*) promotes MET *via* the activation of the epidermal growth factor receptor (EGFR) signaling pathway [93].

Cancer cells have the ability to activate the oncogenic pathways that promote cell proliferation in the local niches, followed by the recruitment of EVs, in particular the exosomes, to communicate with cells at distant sites. The aberrations in the pathways within the cells receiving these EVs can result in cancer progression [94]. Interestingly, the exosomal contents promote EMT by regulating EMT-associated signaling pathways such as the Hippo pathway, β-catenin signaling pathway, and ERK pathway [81]. In breast cancer cells, exosomes obtained from mesenchymal stem cell differentiated adipocytes induced EMT via the activation of YAP and TAZ, the downstream effectors of the Hippo pathway [95]. In prostate cancer, *miR-301a* promoted EMT, via the Wnt/β-catenin pathway, by inhibiting *p63* thereby releasing *ZEB1/2* which suppresses *E-cadherin* [96]. Similarly, *miR-1260b* derived from lung cancer exosome promoted EMT via the Wnt/β-catenin pathway by suppressing SMAD4 and sFRP1 [97]. In non-small lung cell cancer (NSLCC), *miR-92a* promoted EMT through the PI3K/AKT pathway by inhibiting *PTEN* expression [98]. In hepatocellular carcinoma, exosomes induced EMT by activating the TGF- β/Smad signaling pathway, thereby promoting migration and invasion by down-regulating *E-cadherin* expression and up-regulating *Vimentin* expression [99]. Furthermore, melanoma cell-derived exosomes induced EMT via the upregulation of *Let7i*, *let7a*, *miR-191*, *SNAIL2*, *ZEB2*, and *vimentin* and downregulation of *E cadherin* [100].

## Exosomes regulate EMT in EOC

Previously, ascites-derived exosomes enriched in *miR-6780b-5p* were shown to promote EMT by transferring the miRNA to OC cells [31]. Similarly, TGFβ1 within the exosomes derived from cancer-associated fibroblast (CAF) promoted EMT by activating the SMAD signaling pathway [101]. In addition, a previous finding revealed higher levels of EMT markers in A2780 OC cells, exogenously engineered to express SMAD4 mutations, upon treatment with carboplatin [102].

To demonstrate the ability of exosomes to change cells' characteristics and phenotype, we swapped propagation conditioned media between the low-grade EOC cell line (MCAS) and borderline EOC cells (OSE2). We subsequently monitored the change in expression levels of the mesenchymal markers (*N cadherin* and *ZEB1*) and epithelial markers (*E cadherin*). The results indicated downregulation of *N-cadherin* and *ZEB1* markers in MCAS cells treated with media from OSE2, while OSE2 cells treated with media from MCAS cells displayed higher levels of *N cadherin* and *ZEB1*. However, no significant changes were observed in *E cadherin* expression. Similarly, treatment of normal ovarian cells (HOSE 6-3) with media from the high-grade EOC cells (OVSAHO) altered the expression of EMT markers.

Interestingly, the mesenchymal marker expression levels, *SNAI1*, were higher in HOSE 6-3 cells treated with media (exosomes) from OVSAHO cells. Conversely, the expression was lower in OVSAHO cells subjected to exosomal enriched media from HOSE cells 6-3. Moreover, *E cadherin* expression was up-regulated in OVSAHO cells treated with media from HOSE 6-3. These results underline the importance of exosomes in reverting cells' status from transformed to normal cells or vice versa.

## Exosomes as biomarkers for cancer

Due to the presence of exosomes in the body fluids, their potential as a carrier of biomarkers is ideal, allowing a noninvasive liquid biopsy technique to diagnose diseases such as cancer [35,103]. The levels of exosomes are higher in the circulation of tumor patients compared with healthy individuals indicating the association between carcinogenesis and higher exosome secretion [104]. In order to classify the tumor type, genomic profiling of the exosome appears to be a potential biomarker source [41]. Several studies have shown that the tumor-specific factors expressed in the tumor-derived exosomes [105], such as the elevated levels of specific proteoglycan in pancreatic cancer-derived exosomes, may serve as a noninvasive biomarker for early-stage cancer detection [106]. Hence, the cargos loaded within the exosomes may show clinical relevance to tumor progression, and detecting the EV could potentially affect cancer diagnosis and prognosis [107]. Recent findings have emphasized the role of dysregulated exosomal noncoding RNAs (ncRNAs) in cancer development by facilitating cell proliferation, metastasis, immune suppression, and angiogenesis [108–110], thereby having a biomarker role [111].

ncRNAs are a nonprotein-coding class of RNAs that interact with DNA, RNA, or protein to control the expression of genes at transcriptional, post-transcriptional, and epigenetic levels [112]. miRNAs are short ncRNAs of 22 nucleotides in length that play a crucial role in silencing the genes at the post-transcriptional level by binding to the 3′ untranslated region of the target mRNA [113–115], while lncRNAs are a subtype of ncRNAs that are more than 200 nucleotides in length [116]. Numerous studies have elaborated the functions of ncRNAs, such as miRNAs, piRNAs, siRNAs, and lncRNAs, in genome stability, chromatin modifications, gene regulation, as a key regulator of epigenetic control [112], and the aberrant expression of these ncRNAs have been linked with the development of several diseases, such as cancer [117]. Furthermore, the transcriptional regulators and RNA molecules enriched within the exosomes are known to induce an epigenetic change and alter the phenotype of the target cells [118]. Hence, detecting the epigenetic markers within the exosomes could play a significant role in cancer diagnosis and prognosis [119].

Exosomal miRNAs and lncRNAs play a key role in cancer due to their ability to regulate tumor growth, angiogenesis, and drug resistance [116]. Exosomal miRNAs have been commonly exploited as noninvasive cancer diagnostic markers [120] due to their critical role in the occurrence and progression of cancer [121] as well as their higher stability and existence in bodily fluids [121]. Similarly, lncRNAs have a crucial role in several biological processes, including carcinogenesis, by altering gene expression. Recent studies highlight the function of exosome derived lncRNA in regulating tumor apoptosis [122], cancer cell proliferation, migration and angiogenesis [123], thereby serving as diagnostic and prognostic markers in several cancers.

A previous study reported that exosomal *miRNA-21* was overexpressed in the serum of esophageal squamous cell cancer patients compared with benign disease and was found to correlate with tumor progression and aggressiveness, suggesting its role as a biomarker [124]. In prostate cancer, *miRNA-375*, *miRNA-141*, and *miRNA-107* were found to be upregulated, suggesting their role as a diagnostic marker [125]. Exosomal *miRNA-200* regulates gene expression and EMT and promotes metastasis in breast cancer [126]. Exosomal *miRNA-146a* facilitates cancer cell proliferation, and survival in pancreatic cancer [127]. Furthermore, tumor-released exosomes enriched with *miR-21* caused an increase in vimentin and a decrease in E-cadherin, enhancing metastasis via the EMT pathway [128,129]. Singh *et al.* demonstrated that exosomes enriched with *miR-10b*, a miRNA produced by metastatic breast cancer cells, could be transferred from MDA-MB-231 cells to noncancerous HMLE cells through the transfer of exosomes, thereby inducing invasion in the recipient cells [130]. Hence, tumor cells with lower invasive potential could become more aggressive by obtaining exosomes enriched with miRNAs from highly invasive tumor cells, indicating the role of exosomes in influencing the tumor microenvironment [130].

Several studies implicate lncRNAs as cancer-associated molecules enriched within exosomes and have higher tissue specificity than protein-coding mRNA, thereby representing a powerful biomarker [131]. lncRNAs packed within the exosomes serve as a messenger for cell–cell communication to control tumor growth, angiogenesis, and metastasis and modify the tumor microenvironment [132,133]. A previous study showed that lncRNA were exchanged between the gastric cancer cells via the exosomes, thereby participating in cancer progression [134]. lncRNA *FAL1* was overexpressed in the serum exosome of hepatocellular carcinoma tissues and promoted HCC cell proliferation and migration by acting as a sponge of *miR-1236* [135]. lncRNAs-*ATB UCA1* was found to be significantly higher in the exosomes secreted by the tamoxifen-resistant LCC2 breast cancer cells. Exosome-mediated transfer of *Zinc Finger Antisense 1* (*ZFAS 1*) lncRNA enhanced GC cell proliferation and migration by promoting cell cycle progression and EMT [134]. Furthermore, MCF-7 cells, when treated with these LCC2 secreted exosomes enriched in *UCA1*, resulted in higher cell viability, a reduced cleaved caspase3 expression and reduced apoptosis following tamoxifen treatment, suggesting increased tamoxifen resistance [136].

## Exosomal miRNAs & lncRNAs as tumor biomarkers in EOC

Several miRNAs are reported to be commonly altered in EOC patients, suggesting further investigation of their role as candidate biomarkers for early EOC detection (see Table 2) [137].

**Table 2.** Exosomal miRNAs and lncRNAs as biomarkers for EOC diagnosis.

Biomarker type	Potential biomarkers	Derived from	Ref.
lncRNAs	*MALAT1*	serum	[138]
	*UCA1*	serum	[139]
	*aHIF*	serum	[140]
miRNAs	*miRNA-200a, miRNA-200b, miRNA-21, miRNA-141, miRNA-203*	serum	[141]
	*miRNA-375, miRNA-1307*	serum	[142]

For example, deregulated expression of eight exosomal miRNAs (*miR-16, miR-21, miR-93, miR-100, miR-126, miR-200b, miR-223*, and *miR-320*) was reported in EOC patients as compared with healthy individuals. Furthermore, elevated expression levels of exosomes were observed in the plasma of EOC patients, particularly FIGO IV patients, compared with healthy women, indicating that higher exosomal release correlated to higher invasive potential [143].

Similarly, several studies implicate lncRNAs as cancer-associated molecules enriched within exosomes (see Table 2) and have higher tissue specificity than protein-coding mRNA, symbolizing robust biomarkers [131]. For example, exosomal long noncoding RNA *MALAT1* expression was found to be higher in metastatic cells and in the released exosomes. Furthermore, exosomal *MALAT1* derived from EOC cells promoted angiogenesis when transferred to the HUVEC via the exosomes. Additionally, *MALAT1* is a promising noninvasive predictive biomarker for EOC prognosis [138]. Similarly, exosomes secreted by SKOV-3 cells enhanced the migratory and invasive ability of OC cells. One of the suggested mechanisms underlying the cancer progression was the elevated expression of the *focally amplified lncRNA on chromosome 1* (*FAL1*) in OC cells following exosome treatment, which regulated, in turn, the PTEN/AKT pathway [144].

## Involvement of exosomal ncRNAs in chemoresistance of OC cell

One of the main obstacles to the treatment of cancer patients is chemoresistance, with the highest chemoresistance rates observed in OC patients. Recent studies have revealed the role of exosomal miRNA in developing chemoresistance in OC cells [23]. Exosomal transfer of *miR-21* to OC cells promoted resistance to paclitaxel by targeting the *APAF1* gene [145]. Similarly, *miR-433* was observed to cause the downregulation of cyclin-dependent kinase 6 to promote paclitaxel resistance in OC cells [146]. In OC patients, exosomal *miR-223* obtained from hypoxic macrophages induced chemoresistance of OC cells by regulating the PTEN-PI3K/AKT signaling pathway [147].

Similarly, long noncoding RNA *UCA1* was elevated in serum exosomes derived from cisplatin-resistant OC tissues and cell lines compared with normal ovarian samples. Moreover, inverse relation was observed between *UCA1* and *miR-143* in serum exosomes obtained from OC samples. Hence, *UCA1* induced cisplatin resistance in OC by sponging *miR-143*, which resulted in the upregulation of *FOSL2* [139].

## Exosomal proteins as diagnostic markers in cancer

Exosomes contain several proteins such as cytosolic, common membrane proteins, and origin-specific sub-set of proteins that reflect cell function [120]. Numerous studies have shown the potential role of exosomal proteins in serving as a biomarker for various diseases, including cancer [120]. In chronic myeloid leukemia patients, the exosomes derived from the tumor cells carried cytokine TGF-β, which promoted tumorigenesis by activating the AKT, ERK, and anti-apoptotic pathways [148]. In another study, CD63, a member of the tetraspanin family and an exosomal marker, was significantly elevated in melanoma patients compared with healthy individuals [149]. In glioblastoma patients, glioblastoma-specific epidermal growth factor VIII was detected in serum exosomes, serving as a diagnostic tool for glioblastoma detection [150]. A previous study reported the metastatic potential of the exosomes secreted by the glioma cells containing EGFRvIII factor, which promoted the growth of the recipient cells by activating transforming signaling pathways such as mitogen-activated protein kinase and Akt pathway [151]. In breast cancer patients, nephronectin, a protein inducing anchorage-independent growth, was highly expressed in exosomes derived from tumor tissues [152]. Similarly, in the early stage of breast cancer patients, survivin levels were higher in the serum exosomes, suggesting its potential use as a diagnostic and prognostic marker [153].

In EOC, exosomal proteins play a role in tumor growth and metastasis [28]. A previous finding on the content of IGROV-1 and OVCAR-3-derived exosomes showed the presence of proteins involved in signal transduction, adhesion, immune response regulation, and membrane transport and fusion. Liang *et al.* showed the enrichment of cancer-associated signaling pathway proteins within the exosomes such as epithelial cell surface antigen (EpCAM), epidermal growth factor receptor (EGFR), apolipoprotein E (APOE), proliferation cell nuclear antigen (PCNA), with a potential role as diagnostic markers and therapeutic targets for OC treatment [154]. In the study of Marta Szajnik *et al.*, exosomal protein content was higher in advanced stages than in the early stages of OC. Furthermore, TGF-β and MAGE3/6 could be used as markers to distinguish patients with benign tumors from OC patients [155]. Hence, exosomal proteins could reflect the stage of the tumor and serve as a prognostic marker to determine the response to OC treatment [28].

## Exosomes as a therapeutic target

The therapeutic potential of exosomes *in-vivo* has been characterized by emerging techniques such as liquid biopsy, thereby unraveling their clinical importance [156]. Furthermore, exosomes serve as efficient drug delivery vehicles due to their characteristic properties such as low toxicity, low immunogenicity, stability, biocompatibility, permeability, and ability to cross the blood-brain barrier [157,158]. Understanding the constituents, the mechanism of biogenesis and specific cell targeting of exosomes will shed light on their physiological activity. Also, identifying the process regulating the release of the exosomes and their fate in the recipient cells has the potential to unravel novel ways involved in cell–cell communication and inhibition of cancer progression [51].

miRNAs and lncRNAs play a key role in human pathologies such as cancer. Profiling exosomal miRNAs serve as a promising tool for cancer and may be used for monitoring disease recurrence and responses to therapies [78]. Recently, exosomes were found to inhibit tumor angiogenesis when loaded with tumor suppressor miRNA, which acts against pro angiogenetic mRNAs [29]. Similarly, exosomes produced from self-derived dendritic cells were loaded with siRNA and injected into mice, which, in turn, delivered the siRNA to neurons, oligodendrocytes, and microglia in the brain. This resulted in the knockdown of a gene involved in Alzheimer's disease, demonstrating nanovesicle-mediated siRNA delivery's therapeutic use [159]. Furthermore, exosomes enriched with *miRNA-155* inhibitor were successfully delivered to the mouse hepatocytes resulting in the downregulation of TNF in the recipient cells, demonstrating its application in nanomedicine [160].

Several reports have identified the ability of exosomes to precisely deliver drugs to the tumor cells, thereby enhancing the efficacy of cancer treatment. Kim *et al.* demonstrated 50-times higher cytotoxicity in drug-resistant cancer cells upon treatment with paclitaxel-loaded exosomes compared with paclitaxel alone [161]. Similarly, exosomes derived from siGRP78 transfected bone marrow mesenchymal stem cells in hepatocellular cancer inhibited sorafenib resistance, growth, and metastasis *in-vivo* [162]. In triple-negative breast cancer, exosomes engineered with surface peptides against the mesenchymal-epithelial transition factor gene displayed efficient cellular uptake and increased tumor targeting effects of doxorubicin [163]. Similarly, exosomal enclosed curcumin promoted apoptosis in brain cells upon intranasal administration [164].

## Conclusion

Exosomes have proved to be an attractive tool for scientists to examine the diversified roles in the transduction of different contradictory signals based on the information they carry. They are suspected of playing crucial roles in EMT, promoting metastasis, cell survival, and drug resistance. Interestingly, recent data pinpoint exosomes as potential biomarkers for several diseases, including cancer of the ovary. This can be an essential step in improving therapies and managing conditions.

## Future perspective

One of the reasons for the failure of EOC treatment is the detection of the disease at advanced stages in addition to either ineffective drug delivery to the tumor site or acquired resistance to chemotherapy. Therefore, biological markers with the potential to detect EOC at early stages would help improve therapies and overall survival. Exosomes can potentially serve as an effective noninvasive tool as they are carriers of nucleic acids and proteins, which are known to be altered according to the pathological conditions of the patients.

Liquid biopsy, a minimally invasive blood test, is increasingly used for the early detection and prognosis of EOC patients, and circulating exosomes are a great source of liquid biopsy analysis. The circulating exosomal ncRNAs are gaining wide attention as they represent a crucial source of liquid biopsy markers in cancer diagnosis, prognosis, and response to treatment. In addition, exosomes could be utilized in real-time cancer detection, identifying tumor stages and therapeutic responses [165]. Furthermore, exosomes have a promising role in nanomedicine due to their pathophysiological and biological features and functioning as therapeutic targets, anticancer drug delivery vehicles, and diagnostic markers.

Despite the strides in elucidating exosome-associated molecular mechanisms of tumor progression, further efforts are required to utilize the therapeutic potential of exosomes for clinical practice. In addition, further studies on the enormous sample size are required to attain knowledge of its diagnostic value as well as a standardized protocol should be devised for the isolation and characterization of exosomes. Hence, a deeper understanding of the exosome heterogeneity and its use as a drug delivery tool can open new avenues for combating EOC.

**Executive summary**

**Background**

- Exosomes are nanovesicles released in the extracellular milieu by diverse cell types and serve as carriers of proteins, and nucleic acids, with the potential to modulate the recipient cells.
- Understanding the functions of exosomes as messengers between cancer and healthy cells unfolds new avenues in intercellular signaling mechanisms.

**Exosomes as EOC biomarkers**

- Functional characterization of exosomal contents through different techniques opens a new approach to discovering novel EOC biomarkers with the potential to discriminate patients at early stages.
- Several findings have highlighted the involvement of exosome encapsulated genes in EOC angiogenesis, proliferation, migration, and tumor growth, thereby serving as diagnostic and prognostic factors in EOC progression and metastasis.
- Exosomes could represent a powerful tool for therapeutic interventions due to their ability to function as drug carriers or vaccines posing a promising option in curing EOC.
